# A partial oxidation-based approach to the synthesis of gold-magnetite hybrid nanostructures

**DOI:** 10.1038/s41598-024-58145-0

**Published:** 2024-03-28

**Authors:** Rocío A. González Ochea, Tamara B. Benzaquén, Ezequiel R. Encina

**Affiliations:** 1 INFIQC-UNC-CONICET, Departamento de Fisicoquímica, Facultad de Ciencias Químicas, Universidad Nacional de Córdoba, 5000 Córdoba, Argentina; 2 CITeQ (UTN-CONICET), Centro de Investigación y Tecnología Química, Maestro Marcelo López Esq. Cruz Roja Argentina, (5016ZAA), Córdoba, Argentina

**Keywords:** Nanoparticle synthesis, Materials chemistry

## Abstract

Hybrid nanostructures composed of gold and magnetite are of singular interest because they allow the integration of plasmonic and magnetic properties in a single object. Due to this feature, their application has been proposed to perform various functions. The methods usually employed to prepare these particular kinds of nanostructures follow organic phase routes, whereas synthetic methodologies that employ more sustainable solvents have been much less explored. In this work, an environmentally friendly approach for the synthesis of gold-magnetite hybrid nanostructures in aqueous media is proposed. This approach relies on the partial oxidation of the Fe(II) precursor using hydrogen peroxide as the oxidizing agent in the presence of preformed gold nanoparticles dispersed in the reaction medium. The methodology used led to the formation of magnetite nanoparticles with a good stoichiometry and a median size of 30 nm. Furthermore, in the presence of gold nanoparticles in the reaction medium, the formation of gold-magnetite hybrid nanostructures is produced as a consequence of the heterogeneous nucleation of the iron oxide phase on the surface of the gold nanoparticles that act as seeds. The approach reported broadens the possibility of synthesizing hybrid nanostructures in aqueous media with integrated plasmonic and magnetic properties.

## Introduction

Hybrid nanostructures (HNs) composed of materials with different physicochemical properties have been widely investigated in recent years since they allow multiple functionalities to be integrated into a single object, a feature that is difficult to achieve by simply mixing the individual constituents^1^. In particular, special attention has been given to HNs composed of gold (Au) and magnetite (Fe_3_O_4_), as they exhibit plasmonic and magnetic properties that make this particular class of heteronanostructure of interest for potential applications in various fields, such as biomedicine^2–6^, solar energy conversion^7,8^ and environmental remediation^9–11^. Therefore, several methodologies have been developed to prepare NHs composed of gold and magnetite with narrow shapes and size distributions. The synthesis of this class of HNs is generally performed following a seed-mediated approach whereby secondary nanoparticles (NPs) nucleate and grow on the surface of seeds through heterogeneous nucleation^12^. According to the specific experimental conditions, core–shell or Janus morphologies were obtained^13^. The methods for preparing Au-Fe_3_O_4_ HNs can be roughly classified according to the solvent employed. Organic-phase routes are generally based on the thermal decomposition of an organometallic iron precursor on the surface of preformed Au NPs, where HNs are formed through epitaxial growth of the iron oxide phase on the Au seeds. The ratio of the Au seed to the iron precursor, the surfactant amount and the reaction temperature are key experimental variables that must be carefully controlled to ensure that the threshold for the homogeneous nucleation of Fe_3_O_4_ is not reached throughout the synthesis and therefore to boost the heterogeneous nucleation mechanism. In this regard, Sun and coworkers developed a successful methodology to obtain dumbbell-like bifunctional Au-Fe_3_O_4_ HNs, the growth of which was affected by the polarity of the solvent^14^. Shi et al. reported a general thermal decomposition approach using Fe(acac)_3_ as the iron precursor for obtaining Au-Fe_3_O_4_ HNs with diverse morphologies and narrow size distributions^15^. Mezni et al. proposed a one-pot approach using triethylene glycol as a solvent and reducing and stabilizing agent that produces mainly Au-Fe_3_O_4_ HNs with a core–shell morphology^16^. Usually, the materials obtained following these organic phase routes are capped with hydrophobic ligands such as oleic acid or oleylamine, so they exhibit low solubility in water, which limits their potential applications^17^.

The use of less expensive and environmentally friendly solvents has positive implications from both an economic and a sustainability point of view. Therefore, several approaches have been reported for synthesizing Au-Fe_3_O_4_ HNs in aqueous media. The synthesis of NHs in an aqueous medium holds promise for replacing organometallic precursors such as iron pentacarbonyl or iron acetylacetonate by aqueous iron salts such as sulfates, chlorides, and nitrates. These salts are non-volatile and less toxic compared to organometallic precursors. Additionally, there are ongoing investigations into replacing organic solvents and stabilizers used in the organic route, such as diphenyl ether, 1,2-hexadecanediol, benzyl ether, triethylene glycol by water. Moreover, these alternative methodologies allow for mild reaction conditions, unlike the thermal decomposition route which necessitates high-boiling organic solvents, temperatures exceeding 200 °C, long reaction times, and sophisticated equipment. This approach generally relies on the heterogeneous nucleation and growth of the Au component on the surface of preformed Fe_3_O_4_ NPs that act as seeds, resulting mostly in HNs with Janus or core-satellite morphologies^18^. For instance, Zeng et al. proposed a general seed-mediated approach to the synthesis of Au-Fe_3_O_4_ heterodimers in aqueous media by taking advantage of the reductive nature of Fe_3_O_4_ to initiate the reduction of metal cations and deposition of a metal seed on the surface of each Fe_3_O_4_ NP before the metal seed is grown to the desired size through a seed-mediated process^19^. Xing et al. reported a similar two-step method for synthesizing Au-Fe_3_O_4_ HNs, which consists of the reduction of HAuCl_4_ by citrate in the presence of citrate-functionalized Fe_3_O_4_ NPs^20^. However, in contrast with the numerous efforts made to develop methods following organic routes, studies reporting approaches for the synthesis of Au-Fe_3_O_4_ HNs in aqueous media using the Au component as a seed are scarce in the literature. Although several methodologies have been established for synthesizing iron oxides in aqueous media, coprecipitation and partial oxidation methods are the most commonly used methods for the preparation of Fe_3_O_4_ NPs^21,22^. According to the coprecipitation method, particles with sizes typically approximately 10 nm are obtained^23^. On the other hand, following the partial oxidation method, cubic nanometric crystals (30–100) nm or larger spherical particles (0.4–1.1) µm can be obtained depending on the experimental conditions, as O_2_ or nitrate ions are the oxidizing agents usually employed^24,25^. In this process, the initial ferrous hydroxide is transformed into magnetite by means of a dissolution–oxidation–precipitation mechanism^26^. Increasing industrial-scale production of nanostructures could raise concerns about their environmental impact. Comprehensive assessments would be required to address their proper handling, storage and disposal to maximize their benefits and minimize any potential negative impacts, such as the release of nanoparticles into the environment and their potential effects on ecosystems. In this context, the industrial production of nanostructures in aqueous media is highly convenient.

Herein, a partial oxidation-based approach for the synthesis of Au-Fe_3_O_4_ HNs in aqueous media is proposed. This method relies on the partial oxidation of the Fe(II) precursor using hydrogen peroxide as the oxidizing agent in the presence of Au NPs dispersed in the reaction medium. The synthesized materials were characterized by X-ray diffraction (XRD), transmission electron microscopy (TEM), energy-dispersive X-ray spectroscopy (EDS) and UV‒Vis spectroscopy. The concentration of the Fe(II) precursor and the value of the parameter R (R = [(OH)^−^]/[Fe(II)]) were varied to address their effects on the phase, morphology and optical response of the synthesized nanostructures. To achieve deeper insight into the structure‒optical property relationship, extinction spectra were simulated according to the discrete dipole approximation (DDA) method.

## Methods

### Chemicals

Ferrous sulfate heptahydrate (FeSO_4_·7H_2_O, Cicarelli), sodium hydroxide (NaOH, Cicarelli), hydrogen peroxide (H_2_O_2_, Anedra), sodium citrate (Na_3_C_6_H_5_O_7_, Anedra), and tetrachloroauric acid trihydrate (HAuCl_4_·3H_2_O, Sigma‒Aldrich), high purity grade reagents, were used as received without further purification. Ultrapure water with a resistivity of 18.2 MΩ cm was used for the preparation of all aqueous solutions.

### Synthesis of Fe_3_O_4_ NPs

In a typical experiment, the following substances were added sequentially to a three-necked round-bottom flask containing a given amount of FeSO_4_·7H_2_O under continuous bubbling of N_2_(g): 50 mL of a deoxygenated NaOH solution, 50 mL of deoxygenated water, and 50 mL of a deoxygenated H_2_O_2_ solution. After all the solutions were added to the reaction vessel, the mixture was left at boiling temperature for an hour under continuous bubbling of N_2_(g). Prior to mixing the reactants, the dissolved oxygen was removed by bubbling N_2_(g) through the respective solutions for 30 min. The mass of FeSO_4_.7H_2_O was attained at the end of the mixing process at concentrations of 5 × 10^–5^ M or 1 × 10^–4^ M. The initial NaOH concentration was adjusted to obtain values of parameter R of 1.3, 2, and 3 after all the substances were added. The H_2_O_2_ concentration of the 50 mL of deoxygenated solution was adjusted to 5 × 10^–5^ M or 1 × 10^–4^ M depending on whether the final FeSO_4_ concentration was 5 × 10^–5^ M or 1 × 10^–4^ M, respectively. The rate of addition of the solutions was 100 mL/s, unless otherwise specified. A schematic illustration of the experimental setup employed is provided in Supplementary Fig. S1.

### Synthesis of Au NPs

Au NPs were synthesized according to Turkevich’s method using sodium citrate as the reducing agent and HAuCl_4_ as the Au precursor^27^. In particular, the Au NPs were prepared by adding 4.1 mL of a 0.0254 M HAuCl_4_ solution and 10.0 mL of a 9.95 × 10^–3^ M sodium citrate solution to 400 mL of boiling water under magnetic stirring. The heating was turned off when the appearance of the typical ruby-red color was observed after approximately 15 min of reaction. The obtained Au NPs were purified by centrifugation at 1790 RCF for 30 min, after which the pellets were dispersed in ultrapure water. Based on the Lambert–Beer law, the concentration of the purified Au NP suspension was estimated to be 1 × 10^–12^ M (see the Supplementary Information and Supplementary Fig. S2 for further details).

### Synthesis of Au-Fe_3_O_4_ HNs

The procedure for the synthesis of Au-Fe_3_O_4_ HNs was the same as that described above for Fe_3_O_4_ NPs but 50 mL of deoxygenated water was replaced with 50 mL of a deoxygenated 1 × 10^–12^ M Au NP suspension.

### Characterizations

The XRD instrument employed was a PANalytical-X’Pert Pro X-ray diffractometer using Cu Kα radiation (λ = 1.5406 Å). The diffractograms were obtained in the range of 7° − 70° from powders of the particles. To obtain the respective powders, the suspensions containing the reaction products were washed three times with ultrapure water to remove salts formed by counterions such as Na_2_SO_4_, and the precipitated particles were obtained by depositing droplets containing the nanostructures over a glass slide and evaporating the solvent in a fume hood at room temperature. The crystallite size was determined by applying the Scherrer equation to the (220), (311), and (400) reflections; instrumental broadening was considered to be equal to 0.02. TEM images of the prepared nanostructures were obtained by using a JEOL 1120 EXII JEM under an accelerating voltage of 80 kV, whereas the HAADF-STEM and HRTEM images were obtained by using a JEOL JEM-2100 plus under an accelerating voltage of 200 kV. The samples were prepared by adding one drop (~ 30 μL) of the colloidal dispersion onto a holey carbon-formvar-coated copper TEM grid (100 mesh). The analysis of the chemical composition of the synthesized nanostructures was performed by EDS (Oxford). The extinction spectra were measured at room temperature using a Shimazdu UV-1700 Pharma Spec spectrophotometer in the range of (300–900) nm with a cuvette with a 1 cm optical path length.

### Computational methods

The extinction efficiency spectra of the Au NPs, Fe_3_O_4_ NPs and Au-Fe_3_O_4_ HNs were simulated using both the discrete dipole approximation (DDA) method and Mie theory. The DDA method is implemented in the DDSCAT 7.3 code^28^. Further details about this methodology can be found elsewhere^29,30^. A lattice spacing (interdipole distance) of 1 nm was set in all the simulations to model the Au NPs, Fe_3_O_4_ NPs and Au-Fe_3_O_4_ HNs. In previous work, we have shown that this interdipole distance is small enough to achieve precise results^31^. Simulations based on the Mie theory, which is implemented in the BHMIE code^32^, were performed to further verify that a lattice spacing of 1 nm allows us to reach an acceptable error tolerance. The wavelength-dependent dielectric constants of Fe_3_O_4_ and Au were obtained from the literature^33,34^. The refractive index of the environment was set to 1.33, corresponding to water. To simulate the extinction efficiency spectra, the median sizes of the Fe_3_O_4_ NPs and Au-Fe_3_O_4_ HNs were obtained from the statistical analysis of the TEM images.

## Results and discussion

**Figure 1:**
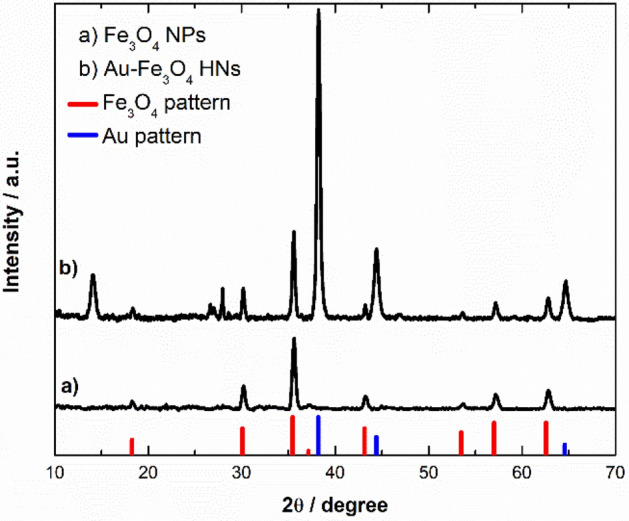
XRD patterns of (**a**) Fe_3_O_4_ NPs and (**b**) Fe_3_O_4_-Au HNs. The reference XRD patterns of Fe_3_O_4_ (JCPDS 01-089-0691) and Au (JCPDS 03-065-2870) are shown in the lower parts as red and blue bars, respectively. The patterns have been arbitrarily shifted in the y-axis.

The crystalline phase of the synthesized nanostructures was characterized through XRD measurements. Figure 1, curve a, shows the XRD pattern of the material obtained for R = 2 and [Fe(II)] = 1 × 10^–4^ M. For comparison, the reference data of Fe_3_O_4_ (JCPDS 01–089-0691) are shown at the bottom as red bars. The diffraction pattern of the obtained material (curve a) shows peaks at 2θ = 18.3, 30.2, 35.6, 37.3, 43.2, 53.7, 57.2, and 62.8, which are assigned to the reflections of the (111), (220), (311), (222), (400), (422), (511), and (440) planes of Fe_3_O_4_, respectively. In addition, no diffraction peaks attributed to other iron oxide or oxyhydroxide phases are observed. The average crystallite size D of the synthesized Fe_3_O_4_ was calculated by applying the Scherrer equation to three diffraction peaks (2θ = 30.2, 35.6, and 43.2), resulting in a D value of (21.9 ± 0.4) nm. The calculation of the unit cell parameter resulted in a value of a = (8.3832 ± 0.0008) Å, which agrees well with the tabulated value (a = 8.396 Å). The calculated a value not only supports the identification of the iron oxide phase formed but also allows us to estimate the stoichiometry. In general, partially oxidized or nonstoichiometric magnetite can be expressed as Fe_3-δ_O_4_, where δ = 0 corresponds to stoichiometric magnetite and δ = 1/3 corresponds to maghemite (γ-Fe_2_O_3_, tabulated unit cell parameter value a = 8.347 Å)^35^. In this regard, Yang et al. reported that the unit cell parameter a decreases by 0.20 Å per vacancy δ^36^. Considering this relationship, a value of δ = 0.064 was estimated for the synthesized material, whose unit formula might be Fe_2.936_O_4_. These results indicate that under the abovementioned experimental conditions, the partial oxidation method implemented using H_2_O_2_ as the oxidant leads to Fe_3_O_4_ NPs with good stoichiometry as the only reaction product.

**Figure 2:**
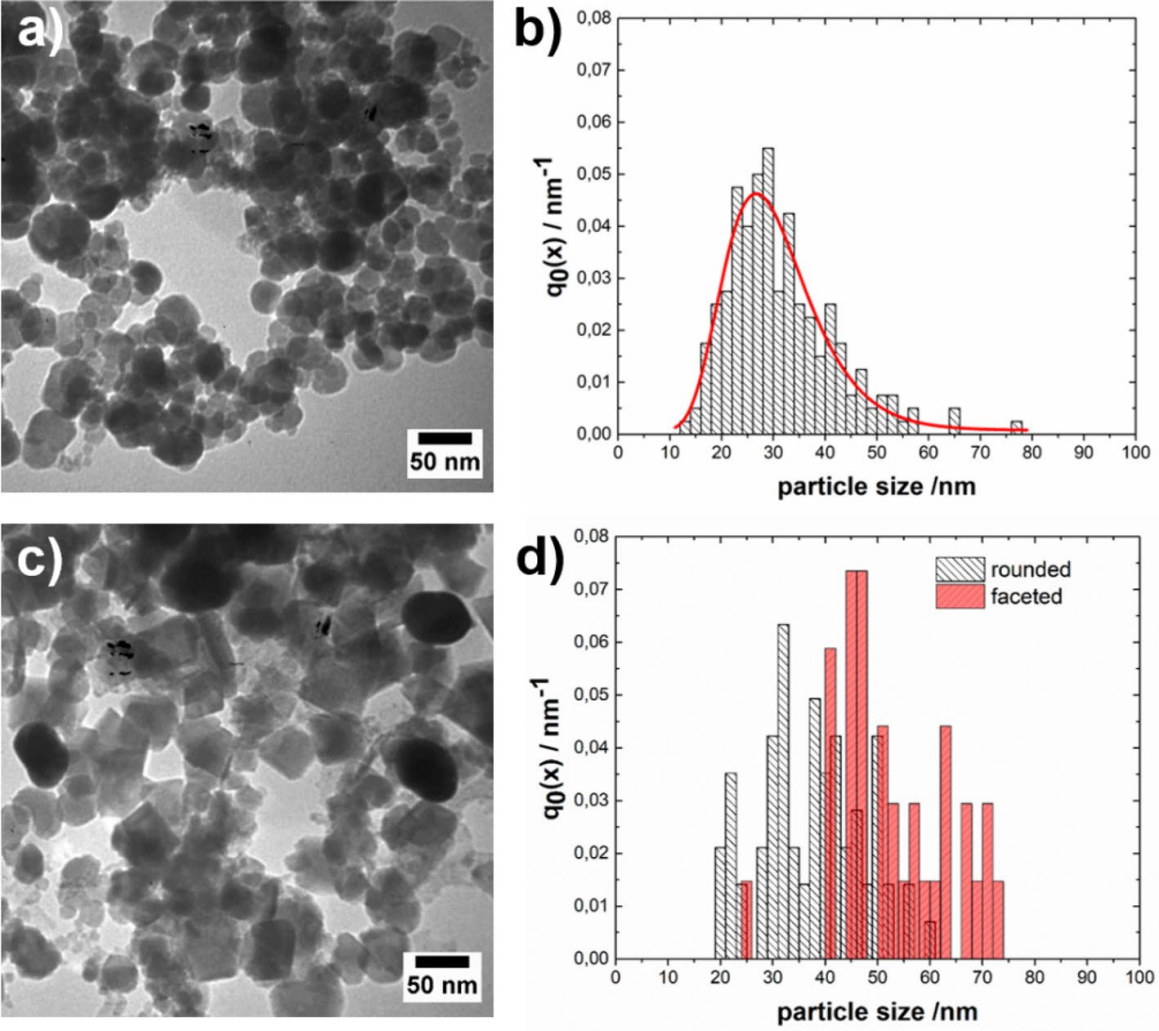
(**a**,**c**) Representative TEM image of the synthesized (**a**) Fe_3_O_4_ NPs and (**c**) Au-Fe_3_O_4_ HNs. (**b**,**d**) Number density distribution of the size, q_0_, of the (**b**) Fe_3_O_4_ NPs and of the (**d**) Fe_3_O_4_ component in the Au-Fe_3_O_4_ HNs.

Figure 2a shows a representative TEM image of the synthesized material, where it can be appreciated that the Fe_3_O_4_ NPs exhibit a rounded quasispherical shape with a rather broad size distribution. The fitting of the number density distribution of the size, q_0_, of the Fe_3_O_4_ NPs to a log-normal distribution revealed that the median size was (30 ± 9) nm (Fig. 2b, red curve). The synthesis of Fe_3_O_4_ NPs with a mean size centered at approximately 30 nm has already been reported when using an aging-based approach employing nitrate as an oxidizing^37^. Interestingly, magnetic particles in the size range of (20–50) nm are of great interest in several fields, such as hyperthermia applications^38,39^. It has been proposed that the transformation of Fe(OH)_2_ into magnetite occurs through a mechanism that involves dissolution of Fe(OH)_2_, oxidation of Fe(II) species in solution and subsequent precipitation of Fe_3_O_4_^40^. Some authors state that primary particles of Fe_3_O_4_ nucleate heterogeneously on the surface of Fe(OH)_2_^41^. A comprehensive study published by Sugimoto and Matijevic on the formation of Fe_3_O_4_ by means of the partial oxidation of Fe(OH)_2_ by KNO_3_ at elevated temperatures revealed that an excess of Fe(II) in the reaction medium led to the formation of relatively large spherical particles (0.4–1.1) µm, whereas an excess of OH^-^ resulted in the formation of smaller cubic particles (30–100) nm^42^. The different particle sizes and morphologies were attributed to distinct mechanisms of growth, which are determined by the pH of the system in relation to the isoelectric point of magnetite. Under experimental conditions where there is an excess of Fe(II) with respect to the stoichiometric quantity necessary for the precipitation of Fe(OH)_2_, the pH of the system is maintained close to the isoelectric point of Fe_3_O_4_, allowing the growth of particles by aggregation and recrystallization of smaller primary particles. In contrast, an excess of OH^-^ keeps the pH of the system above the isoelectric point, causing the charge of the Fe_3_O_4_ surface to prevent its aggregation and producing the growth of the particles due to the addition of species in the solution.

**Figure 3:**
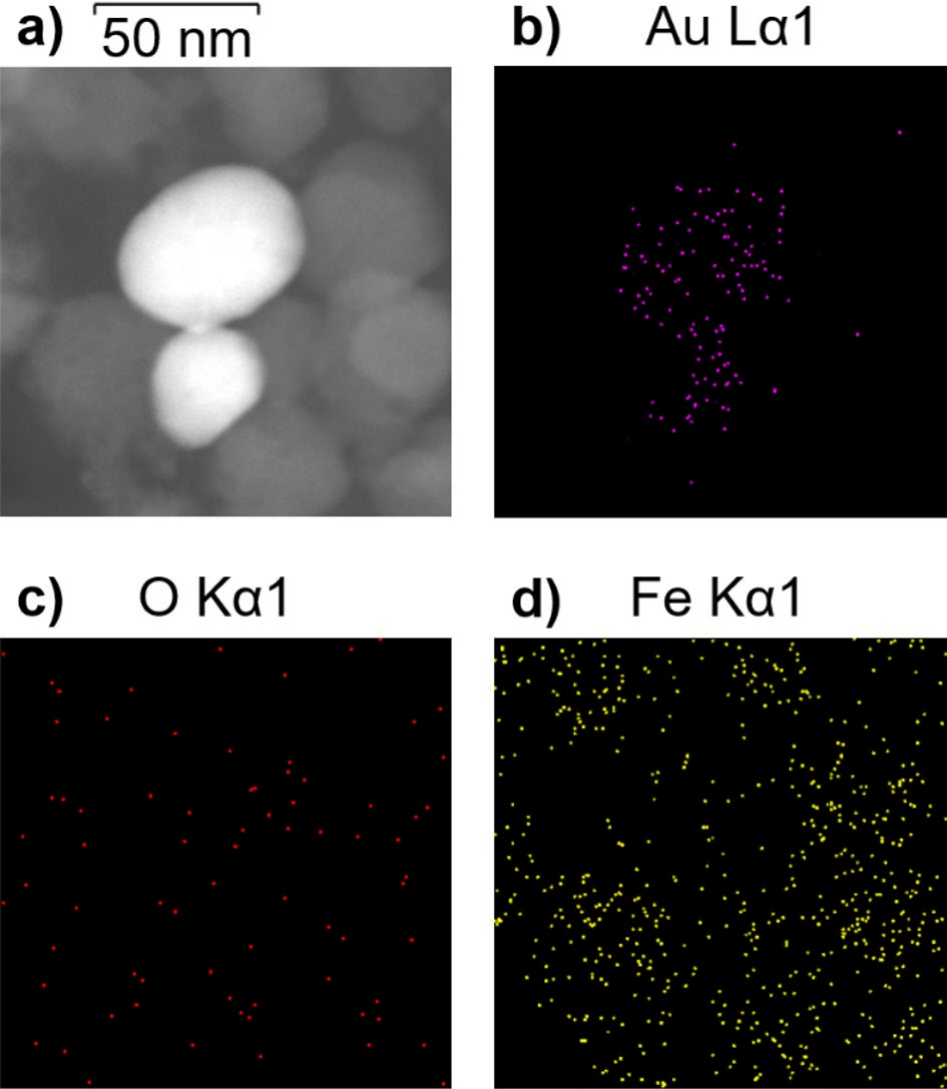
(**a**) HAADF-STEM image and EDS element mapping images for (**b**) Au, (**c**) O, and (**d**) Fe of the synthesized Fe_3_O_4_-Au HNs. The scale bar applies for all images.

More recently, it has been reported that even in the presence of excess OH^-^, the crystallization of Fe_3_O_4_ occurs through the formation and aggregation of primary particles^43^. In addition to crystallization via particle attachment, faceted magnetite crystals were found, indicating that coarsening also occurs to minimize the total energy of the developing crystals. The difference found between the average crystallite size D (21.9 ± 0.4) nm and the median Fe_3_O_4_ NP size obtained through statistical analysis based on the TEM images (30 ± 9) nm suggested that under the experimental conditions, the crystallization of Fe_3_O_4_ might occur through the formation and aggregation of primary particles. Based on these findings, we implemented a strategy for the synthesis of Au-Fe_3_O_4_ NHs in an aqueous medium that consists of adding Au NPs to the reaction system, considering that the heterogeneous nucleation of Fe_3_O_4_ primary particles might also take place on the Au surface. Figure 1, curve b, shows the diffraction pattern of the obtained material under the same experimental conditions as those already described but with Au NPs dispersed in the reaction medium. In addition to the peaks assigned to reflections of the Fe_3_O_4_ phase, peaks at 2θ = 38.2, 44.4, and 64.7 are observed, which are assigned to reflections of the (111), (200), and (220) planes of Au, respectively. For comparison, the reference data for Au (JCPDS 03-065-2870) are shown at the bottom as blue bars. Furthermore, there are also peaks at 2θ = 14.1 and 27.0, which are assigned to reflections of the (020) and (120) planes of lepidocrocite γ-FeOOH (JCPDS 01-074-1877), respectively. This analysis indicated that the Fe_3_O_4_ phase was formed along with a detectable amount of a completely ferric phase as a byproduct when Au NPs were present in the reaction medium. Indeed, it has been reported that during the aerial oxidation of Fe(II) sulfate solutions, the precipitation of Fe_3_O_4_ occurs via slow oxidation by air, whereas the precipitation of γ-FeOOH occurs via rapid oxidation by air^44^. The average crystallite size D of the Fe_3_O_4_ component was determined following the same procedure as above, resulting in a D value of (26 ± 2) nm. At first glance, this ~ 20% increase in the average D value could indicate that the incorporation of the Au NPs in the system could have an impact on the formation mechanism of the Fe_3_O_4_ component. Furthermore, the calculation of the unit cell parameter of the iron oxide component resulted in a value of *a* = (8.3891 ± 0.0003) Å, indicating the formation of Fe_3_O_4_ with good stoichiometry. Figure 2c shows a representative TEM image of the synthesized Au-Fe_3_O_4_ composite, where the presence of both Au and Fe_3_O_4_ NPs can be observed as a consequence of the substantial differences in the electronic contrast. In particular, Fe_3_O_4_ NPs mostly exhibit a rounded quasispherical shape, but crystals with clearer facets and corners that resemble a cubic or octahedral morphology can also be observed in a smaller proportion. Note that according to theoretical predictions, the main morphology of magnetite crystals is octahedral^45^. Counting more than one hundred NPs, it could be estimated that the amount of rounded (faceted) structures is 70% (30%). Moreover, Altan et al. reported similar results when using NaNO_3_ as the oxidizing^46^. The number density distribution of the size of the Fe_3_O_4_ NPs (Fig. 2d) shows a broader distribution with respect to that determined for the Fe_3_O_4_ NPs synthesized in the absence of Au NPs. Analysis of the size distribution of each morphology revealed that the size distribution of the rounded particles (centered at ~ 30 nm) resembled that of the Fe_3_O_4_ NPs synthesized in the absence of Au NPs, whereas the size distribution of the faceted NPs was shifted toward larger values (~ 45 nm). These results are consistent with the larger average D value calculated for the Fe_3_O_4_ NPs synthesized in the presence of Au NPs. On the other hand, the Au NPs exhibit a prolate shape with a relatively high degree of sphericity. The fitting of their number density distribution to a normal distribution resulted in a mean size of (57 ± 14) nm (see Supplementary Fig. S3). Figure 2c shows that a significant number of Fe_3_O_4_ NPs do not interact with the Au NPs, whose formation might be due to both homogeneous nucleation of primary particles and heterogeneous nucleation on the surface of the Fe(OH)_2_ platelets that are consumed as the reaction progresses. However, the Au NPs appear to be in close contact with the Fe_3_O_4_ component, suggesting that the latter might also nucleate heterogeneously on the Au surface and grow according to the mechanisms described above. Therefore, the structural characteristics of the obtained material might arise as a consequence of different nucleation mechanisms occurring simultaneously during the course of the reaction. Furthermore, the Au-Fe_3_O_4_ NHs do not seem to be completely coated by Fe_3_O_4_ and present a morphology more related to the Janus type than to the core–shell. Figure 3a shows a representative HAADF-STEM image of the Fe_3_O_4_-Au HNs, while its corresponding chemical composition analysis performed using EDS measurements is shown in Fig. 3b–d. The respective EDS element mapping images prove that the synthesized HNs are composed of Au, O, and Fe, respectively, being the elements composing magnetite homogeneously distributed around the Au NPs.

**Figure 4:**
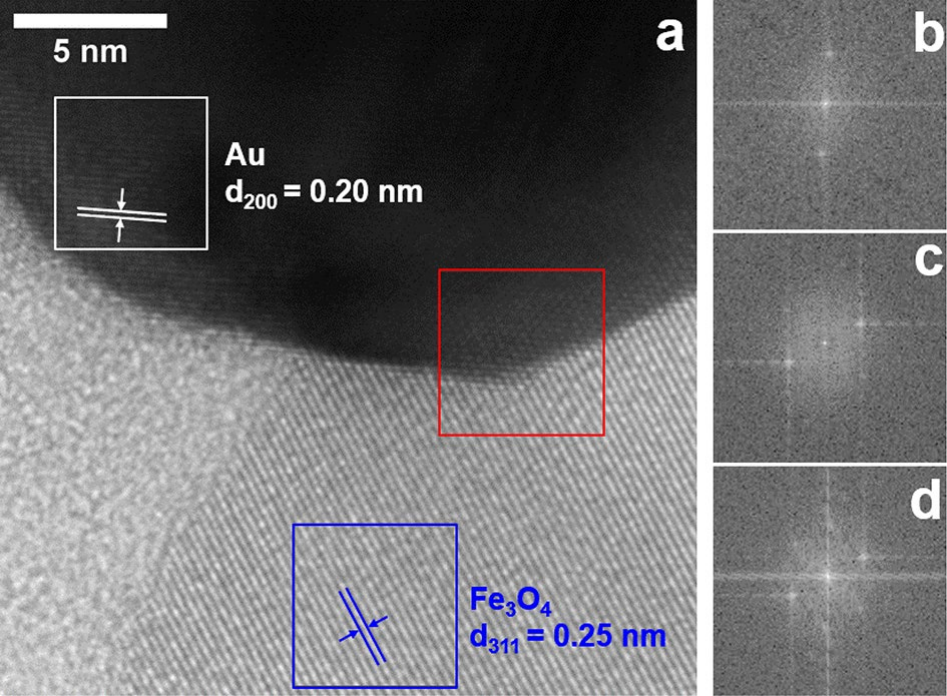
(**a**) High resolution TEM image of Fe_3_O_4_-Au HNs. (**b**–**d**) Fast Fourier transform (FFT) of the rectangular selected area delimited by (**b**) white, (**c**) blue, and (**d**) red lines in panel (**a**), respectively.

The high-resolution TEM image of the Fe_3_O_4_-Au HN, shown in Fig. 4a, illustrates its high crystallinity. Figure 4b,c show the fast Fourier transform (FFT) of the rectangular selected areas delimited by white and blue lines in Fig. 4a, respectively, where a pair of points equidistant from the origin in 1/d can be observed in each case. The interplanar distance was determined by analyzing the respective inverse fast Fourier transform, resulting in a value of 0.20 nm (0.25 nm) for the area delimited by white (blue) lines. Based on the respective JCPDS mentioned above, these values are associated with the family of planes (200) of Au and the family of planes (311) of Fe_3_O_4_. Furthermore, Figs. 4d shows the FFT of the rectangular selected area delimited by red lines in Fig. 4a, that is, the interphase between Au and Fe_3_O_4_, where the spots corresponding to both phases can be observed.

**Figure 5:**
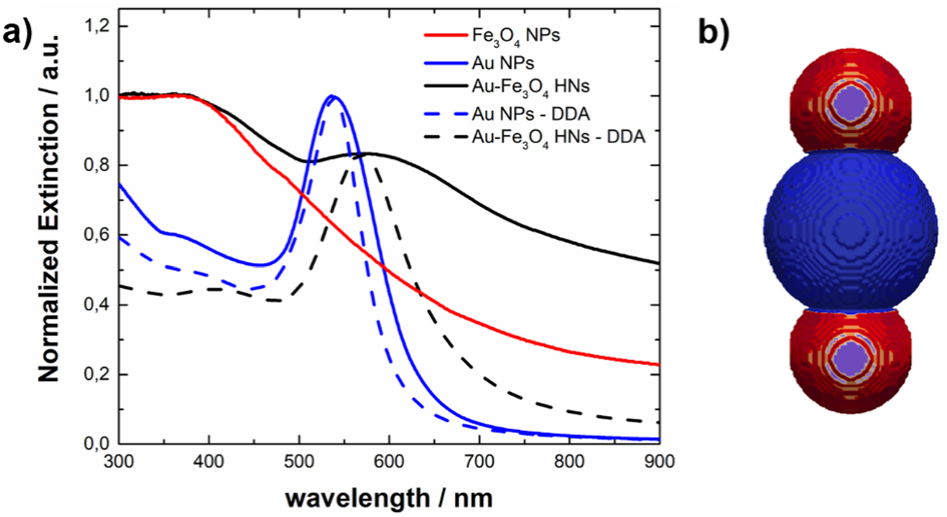
(**a**) Normalized extinction spectra of Fe_3_O_4_ NPs (red curve), Au NPs (solid blue curve), and Fe_3_O_4_-Au HNs (solid black curve) along with the DDA-simulated normalized extinction efficiency spectra of a 57 nm diameter Au nanosphere (dashed blue curve) and a model Au-Fe_3_O_4_ HN schematically depicted in panel (**b**) (dashed black curve). (**b**) Scheme of the model Au-Fe_3_O_4_ HN, which consists of a 57 nm diameter Au nanosphere in contact with two diametrically opposed 40 nm diameter Fe_3_O_4_ spherical caps, with the center of the caps 42 nm displaced with respect to the center of the Au NP.

Colored photos of colloidal dispersions of Au NPs, Fe_3_O_4_ NPs, and Fe_3_O_4_-Au NHs are provided in Supplementary Fig. S4, where it can be appreciated the visual change in the color of the colloidal dispersion after the formation of Au-Fe_3_O_4_ HNs. Figure 5a shows the normalized extinction spectra of the synthesized nanostructures. Note that the concentration of nanostructures is similar in each measured sample. The red curve shows the normalized extinction spectrum of the aqueous dispersion of Fe_3_O_4_ NPs, which exhibits a monotonic decrease in wavelength (λ) > 400 nm and resembles typical spectra previously reported for iron oxide nanoparticles suspended in water^47,48^. The extinction spectra of the Au-Fe_3_O_4_ composite is shown in the solid black curve. A broad peak centered at approximately 575 nm attributed to the localized surface plasmon resonance (LSPR) of the Au component is observed, which seems to overlap with the response of the Fe_3_O_4_ NPs. For comparison, the extinction spectrum of an aqueous dispersion of the Au NPs added to the reaction system for obtaining the hybrid composite is shown as a solid blue curve and is characterized by an LSPR centered at 535 nm. The redshift observed in the LSPR of the Au NPs after the formation of the Fe_3_O_4_ phase is attributed to an increase in the refractive index of the local environment that surrounds the Au NPs, providing evidence of the formation of the Au-Fe_3_O_4_ HNs^49^. These results are in agreement with the morphology of the nanostructures shown in Fig. 2c. To gain deeper insight into the optical properties of the synthesized materials, electrodynamics simulations were performed. Due to the structure–property relationships that are established at the nanoscale, modeling of the extinction spectra provides complementary evidence for the formation of Au-Fe_3_O_4_ HNs.

The dashed blue curve in Fig. 5a shows the DDA-simulated extinction efficiency spectrum of a 57 nm diameter Au nanosphere dispersed in water. Note that Supplementary Fig. S5 compares the spectra calculated through the approximated DDA method and the exact Mie solution; the very good agreement between both spectra indicates that a tolerable error is reached when using an interdipole distance of 1 nm to model the synthesized nanostructures. The spectral position of the LSPR peak in the simulated spectrum matches the wavelength peak in the experimental spectrum of the Au NPs, whereas the broadening of the experimental peak with respect to the simulated peak is attributed to the size and shape distributions. On the other hand, the DDA-simulated extinction efficiency spectrum of the Au-Fe_3_O_4_ HN, schematically depicted in Fig. 5b, whose size and shape approximately resemble those found for the synthesized material, is shown as a dashed black curve. The model Au-Fe_3_O_4_ HN consists of a 57 nm diameter Au nanosphere in contact with two diametrically opposed 40 nm diameter Fe_3_O_4_ spherical caps, with the center of the caps 42 nm displaced with respect to the center of the Au NP. As expected, the LSPR peak for the Au-Fe_3_O_4_ HN was redshifted in comparison to that for the isolated Au nanosphere, and the magnitude of the redshift was similar to that measured for the synthesized Au-Fe_3_O_4_ composite. The experimental peak is notably broader than the simulated peak, which is attributed to the size and shape distributions of the Au-Fe_3_O_4_ HNs. Therefore, this modeling performed considering the morphology and size of the synthesized nanostructures allows us to assign changes in the optical response to the formation of Au-Fe_3_O_4_ HNs. For the sake of clarity, the modeling of the Fe_3_O_4_ NPs is shown in Supplementary Fig. S6, where the accuracy of the DDA results is corroborated by comparison with the Mie theory results. Note that the purpose of performing electrodynamic simulations is to gain insight into the interactions of the components of the of the HN, that is, gold and magnetite. An excellent agreement between the experimental and simulated spectra could be achieved taking into account all the contributions to the extinction of nanostructures of different sizes and shapes present in the sample. However, such a task is beyond the scope of the current work.

**Figure 6:**
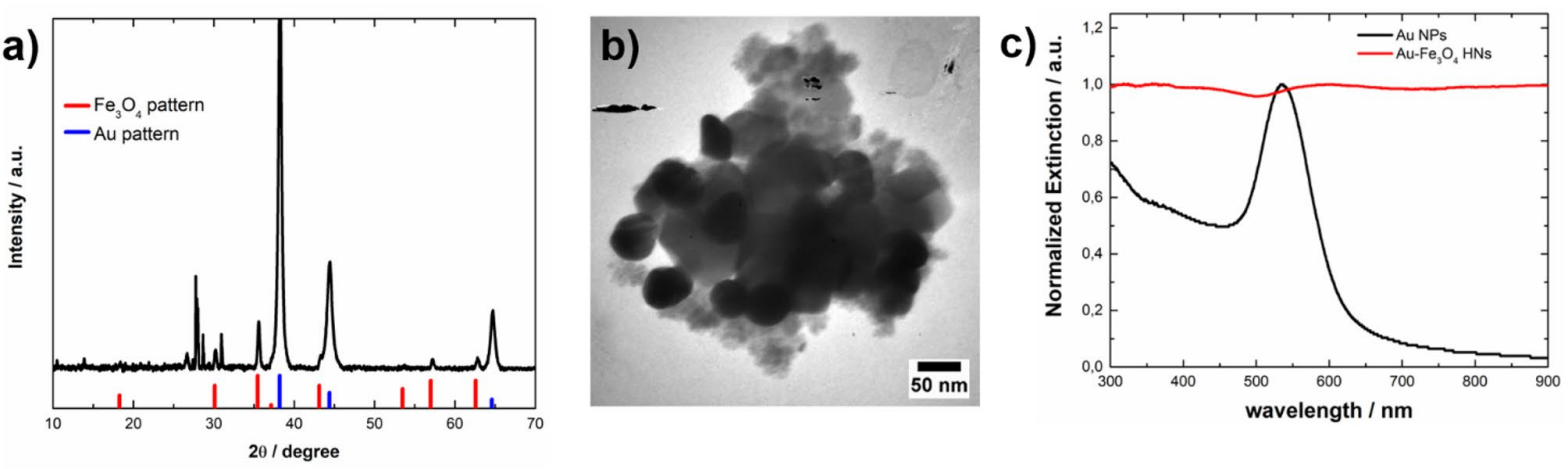
(**a**) XRD pattern, (**b**) representative TEM image, and (**c**) normalized extinction spectrum of Au-Fe_3_O_4_ HNs (red curve) synthesized with R = 1.3. (**a**) The reference XRD patterns of Fe_3_O_4_ (JCPDS 01-089-0691) and Au (JCPDS 03-065-2870) are shown in the lower part as red and blue bars, respectively. (**c**) For comparison, the extinction spectrum of the Au NPs is shown as a black curve.

To address the effects of the parameter R on the phase, morphology and optical response of the synthesized nanostructures, experiments with R = 1.3 and 3 were performed while keeping the concentration of the Fe(II) precursor, the oxidizing H_2_O_2_, and the Au NPs constant. Figure 6a shows the XRD pattern of the material obtained with R = 1.3, where peaks corresponding to reflections of the Fe_3_O_4_ and Au phases can be noted. The peak observed at 2θ = 27.0 is assigned to reflections of the (120) plane of γ-FeOOH, whereas the peak at 2θ = 31.7 is attributed to the (112) plane of Na_2_SO_4_ (JCPDS 01-079-1553), which is formed as a byproduct. A representative TEM image of the synthesized Au-Fe_3_O_4_ composite shown in Fig. 6b reveals the presence of Au NPs attached to the Fe_3_O_4_ nanostructures. The iron oxide composite consists of both larger faceted particles than those found for R = 2 and smaller particles showing a granular morphology. According to previous studies, faceted particles might form due to the accretion of disordered primary particles^50^. The extinction spectrum of the Au-Fe_3_O_4_ NHs synthesized with R = 1.3 (Fig. 6c) shows a flat curve, indicating a significant contribution from the scattering component of the relatively large-sized Fe_3_O_4_ particles (red curve). However, a shoulder centered at 600 nm can also be attributed to the LSPR of the Au component, which is, in turn, redshifted with respect to the spectral position of the LSPR corresponding to the isolated unmodified Au NPs (black curve).

**Figure 7:**
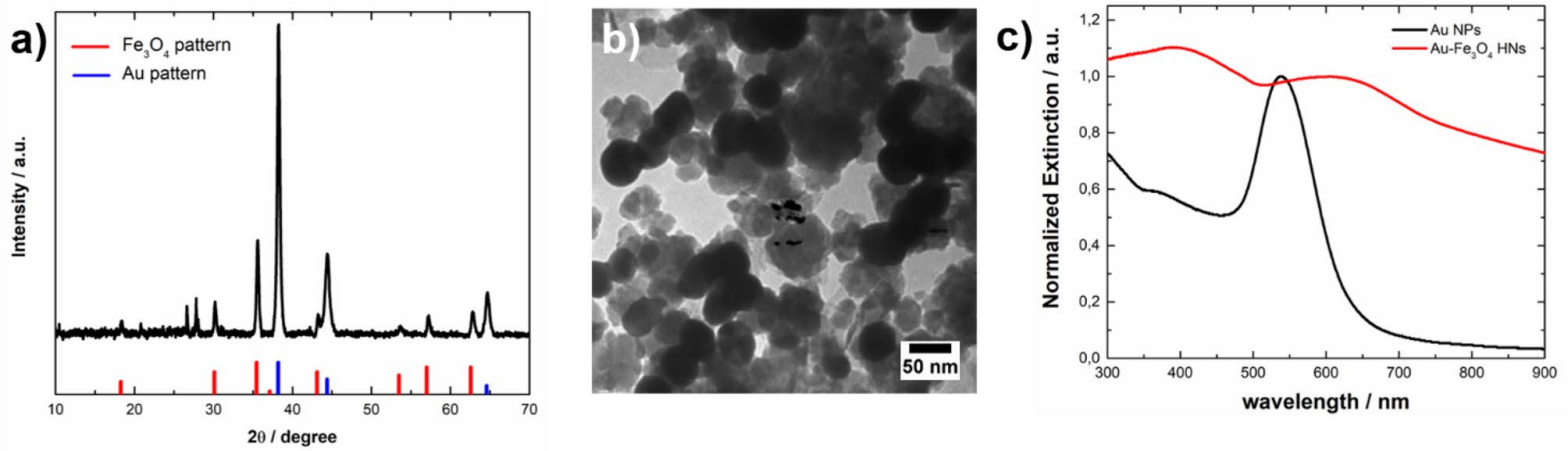
(**a**) XRD pattern, (**b**) representative TEM image, and (**c**) normalized extinction spectrum of Au-Fe_3_O_4_ HNs (red curve) synthesized with R = 3. (**a**) The reference XRD patterns of Fe_3_O_4_ (JCPDS 01-089-0691) and Au (JCPDS 03-065-2870) are shown in the lower part as red and blue bars, respectively. (**c**) For comparison, the extinction spectrum of the Au NPs is shown as a black curve.

The results obtained when using a value of R = 3 for the synthesis of Au-Fe_3_O_4_ NHs are shown in Fig. 7. The peaks in the XRD pattern (Fig. 7a) are assigned to reflections of the phases Au and Fe_3_O_4_, whereas the peak at 2θ = 27.0 is assigned to reflections of the (120) planes of γ-FeOOH. A representative TEM image of the synthesized composite (Fig. 7b) indicated the presence of Au NPs that were in close contact with the rounded Fe_3_O_4_ nanoparticles. The extinction spectrum of the HNs (Fig. 7c, red curve) presents a broad peak centered at 600 nm corresponding to the excitation of the LSPR of the Au component redshifted with respect to the LSPR in isolated Au NPs (see black curve, Fig. 7c). Therefore, the characterization of the obtained materials indicates that Au-Fe_3_O_4_ NHs are also formed when R = 1.3 and 3 are used in the synthesis. For R = 1.3, the size of the Fe_3_O_4_ component is relatively large, which impacts the profile of the extinction spectrum, as expected. In all the cases, a substantial amount of Fe_3_O_4_ possibly formed following a homogeneous nucleation mechanism, which might be associated with excessively high supersaturation values^51,52^.

**Figure 8:**
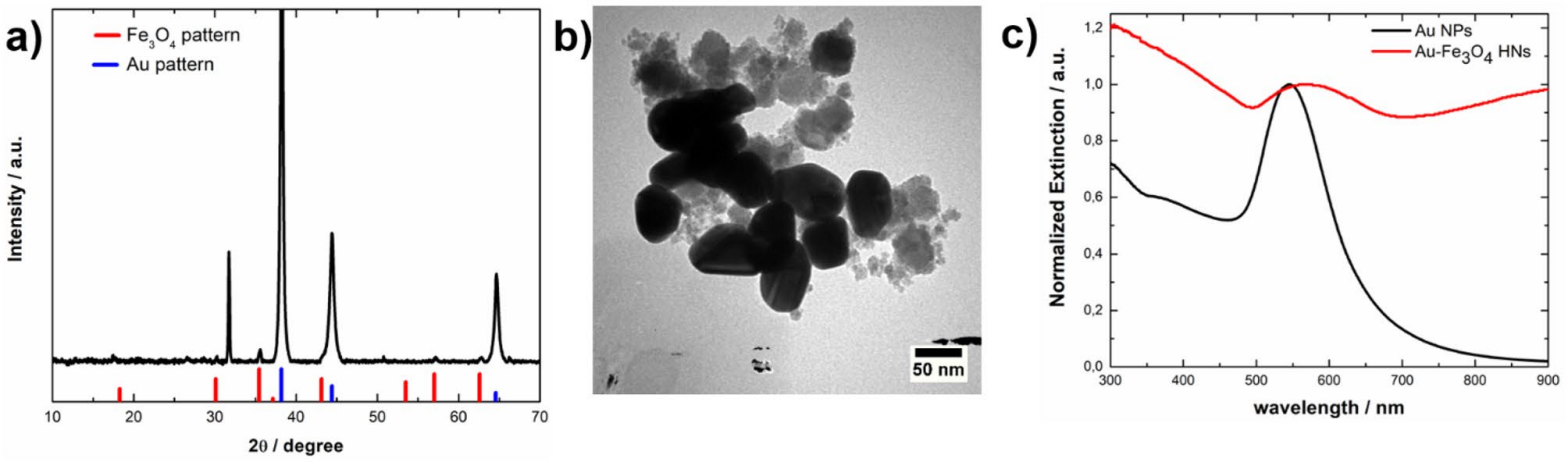
(**a**) XRD pattern, (**b**) representative TEM image, and (**c**) normalized extinction spectrum of Au-Fe_3_O_4_ HNs (red curve) synthesized with R = 2 and a Fe(II) concentration of 5 × 10–5 M. (**a**) The reference XRD patterns of Fe_3_O_4_ (JCPDS 01-089-0691) and Au (JCPDS 03–065-2870) are shown in the lower part as red and blue bars, respectively. (**c**) For comparison, the extinction spectrum of the Au NPs is shown as a black curve.

To reduce the amount of Fe_3_O_4_ possibly formed by a homogeneous nucleation process, additional synthesis experiments were performed in which the Fe(II) concentration was decreased from 1 × 10^–4^ M to 5 × 10^–5^ M using R = 2 and the same Au NP concentration (1 × 10^–12^ M). The results obtained are shown in Fig. 8. The XRD pattern of the synthesized material presents peaks that correspond to reflections of the Fe_3_O_4_ and Au phases, whereas the peak at 2θ = 31.7 is assigned to the reflection of the (112) planes of Na_2_SO_4_, which is formed as a byproduct (Fig. 8a). The representative TEM image shown in Fig. 8b suggests that a lower quantity of Fe_3_O_4_ is formed in comparison with the results obtained using a Fe(II) concentration of 1 × 10^–4^ M, as expected. Furthermore, the amount of Fe_3_O_4_ particles possibly formed by a homogeneous nucleation process seems to be noticeably reduced. In addition, the extinction spectrum presents a broad peak centered at 570 nm attributed to the excitation of the LSPR in the Au-Fe_3_O_4_ NHs (Fig. 8c, red curve). This peak appears to be redshifted with regard to the LSPR peak corresponding to individual Au NPs (see Fig. 8c, black curve) because of the formation of the Au-Fe_3_O_4_ NHs. The increase in the extinction coefficient for λ > 700 nm is mainly due to the coupling between the LSPRs that arises as a consequence of the Au NPs being in contact, as shown in Fig. 8b^53,54^.

**Figure 9:**
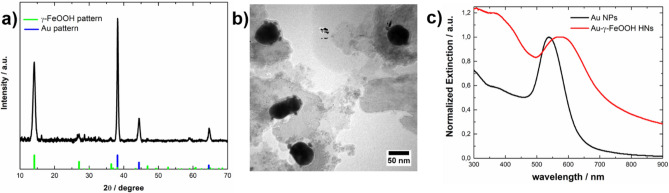
(**a**) XRD pattern, (**b**) representative TEM image, and (**c**) normalized extinction spectrum of Au-γ-FeOOH HNs (red curve) synthesized with R = 2 and a Fe(II) concentration of 5 × 10–5 M. (**a**) The reference XRD patterns of γ-FeOOH (JCPDS 01-074-1877) and Au (JCPDS 03-065-2870) are shown in the lower part as green and blue bars, respectively. (**c**) For comparison, the extinction spectrum of the Au NPs is shown as a black curve.

In all the cases presented thus far, the rate of addition of the H_2_O_2_ solution was 100 mL/s. However, quite interestingly, for an Fe(II) concentration of 5 × 10^–5^ M and in the presence of Au NPs, the iron oxide/oxyhydroxide phase mostly formed can be controlled through the addition rate of the oxidant solution. Figure 9 shows the results obtained when the oxidant addition rate was decreased to 10 mL/s. The XRD pattern of the obtained material presented peaks at 2θ = 38.2, 44.4, and 64.7, which are assigned to reflections of the planes (111), (200), and (220) of Au, respectively, as well as peaks at 2θ = 14.1, 27.0, 36.3, and 46.9 which were assigned to reflections of the (020), (120), (031), and (051) planes of the lepidocrocite γ-FeOOH phase, respectively. Nonetheless, no diffraction peaks attributed to the Fe_3_O_4_ phase are observed. This change in the most common phase produced marked changes in the morphology of the synthesized nanostructures. The representative TEM image displayed in Fig. 9b shows Au NPs with ~ 10 nm particles attached on their surface resembling a core-satellite morphology. Importantly, the HNs appear well separated, indicating that the Au NPs do not interact with each other. Note that for an Fe(II) concentration of 5 × 10^–5^ M in the absence of Au NPs, Fe_3_O_4_ NPs are obtained as reaction products irrespective of the addition rate of the oxidant solution (see Supplementary Fig. S7). Owing to the structure–property relationships that occur at the nanoscale, the morphological features of the synthesized HNs are manifested in their optical response. Thus, the extinction spectrum exhibits a defined peak centered at 575 nm (Fig. 9c) due to the LSPR excitation in the Au-γ-FeOOH NHs, whereas the extinction values rapidly decrease for λ > 650 nm as a consequence of the relatively large separation between the Au NPs that comprise the HNs, preventing the occurrence of plasmonic coupling, which results in extinction increases in the NIR region.

## Conclusions

In this work, a simple and environmentally friendly approach for the synthesis of Fe_3_O_4_ NPs and Au-Fe_3_O_4_ HNs in aqueous media is proposed. This method relies on the partial oxidation of the Fe(II) precursor using H_2_O_2_ as the oxidizing agent. In the absence of Au NPs dispersed in the reaction medium, Fe_3_O_4_ with a good stoichiometry and a median size of 30 nm was obtained as the only reaction product. On the other hand, in the presence of Au NPs dispersed in the reaction medium, Au-Fe_3_O_4_ HNs were obtained as the main reaction product. The morphology of the HNs consisted of approximately 57 nm long quasispherical Au NPs in contact with the Fe_3_O_4_ component, although the HNs were not completely coated by the latter, which resembled the Janus type. The structural features of the NHs were employed to perform electrodynamic modeling of their extinction properties. In particular, the redshift observed in the spectral position of the LSPR was successfully accounted for by means of DDA simulations. The joint analysis of both the experimental and theoretical results suggested that the Fe_3_O_4_ phase nucleated heterogeneously on the Au surface. The results obtained indicate that the value of the parameter R impacts the size of the Fe_3_O_4_ component of the HNs, which in turn modifies their extinction properties. Moreover, a decrease in the concentration of the Fe(II) precursor reduces the amount of Fe_3_O_4_ presumably formed by a homogeneous nucleation mechanism. Furthermore, in the presence of Au NPs, the addition rate of the oxidizing agent seems to play a significant role in the formation of the iron oxide/oxyhydroxide phase. The novel strategy implemented for the synthesis of Au-Fe_3_O_4_ HNs in aqueous media broadens the possibility of integrating plasmonic and magnetic properties into single nanostructures with potential applications in diverse fields.

### Supplementary Information

Supplementary Figures.

## Data Availability

All the data generated and analyzed during this study are included within this published article or its supplementary information.
