# Supplementary material for: A partial oxidation-based approach to the synthesis of gold-magnetite hybrid nanostructures
Source: Sci Rep. 2024 Mar 28;14:7352. doi: 10.1038/s41598-024-58145-0 (PMC10978920; doi:10.1038/s41598-024-58145-0)
Supplement: Supplementary file 1 — Supplementary Figures. [file 41598_2024_58145_MOESM1_ESM.pdf]

Supplementary Information

A Partial Oxidation-Based Approach to the Synthesis of Gold-Magnetite Hybrid Nanostructures

Rocío A. González Ochea[1], Tamara B. Benzaquen[2]

and Ezequiel R. Encina[1,*]

1-INFIQC-UNC-CONICET, Departamento de Fisicoquímica, Facultad de Ciencias Químicas, Universidad Nacional de Córdoba, Córdoba, 5000, Argentina

2-CITeQ (UTN-CONICET), Centro de Investigación y Tecnología Química, Maestro Marcelo López esq. Cruz Roja Argentina, (5016ZAA), Córdoba, Argentina.

* Corresponding author: Ezequiel R. Encina

e-mail address: ezencina@fcq.unc.edu.ar

Tel: +54-351-535-3866   Fax: +54-351-433-4180

This document provides more detailed information to the main paper mentioned above. The following information is included:

Content **Page**

Content	Page
**Figure S1.** Schematic illustration of the experimental setup employed for the synthesis of Fe_3O_4 NPs and Au-Fe_3O_4 HNs.	3
**Figure S2.** Extinction spectrum of the purified Au NPs (black curve) and simulated extinction efficiency spectrum of a D=57 nm Au nanosphere dispersed in water.	4
**Figure S3.** a) Representative TEM image and b) number density distribution of the size, q0, of the Au NPs employed for the synthesis of the Au-Fe_3O_4 HNs. The red curve in panel b) corresponds to a fit to a normal distribution centered at 57 nm.	4
**Figure S4.** Visual change in the color of the colloidal solution after the formation of Au-Fe_3O_4 HNs	5
**Figure S5.** Comparison between the normalized extinction efficiency spectra of a 57 nm diameter Au nanosphere immersed in water calculated through the Mie theory (blue curve) and the approximated DDA method (black dots). The very good agreement between both spectra indicates that a tolerable error is reached when using an inter-dipole distance of 1 nm to model the Au nanosphere.	5
**Figure S6.** Comparison between the normalized extinction efficiency spectra of a 30 nm diameter Fe_3O_4 nanosphere immersed in water calculated through the Mie theory (blue curve) and the approximated DDA method (black dots), where it can be appreciated the high accuracy of the later.	6
**Figure S7.** XRD pattern of Fe_3O_4 NPs synthesized with R=2, Fe(II) concentration $5x10^{-5}$M, and addition rate of H_2O_2 solution of (a) 100 mL/s and (b) 10 mL/s. The reference XRD pattern of Fe_3O_4 (JCPDS 01-089-0691) is shown in the lower part in red bars.	7

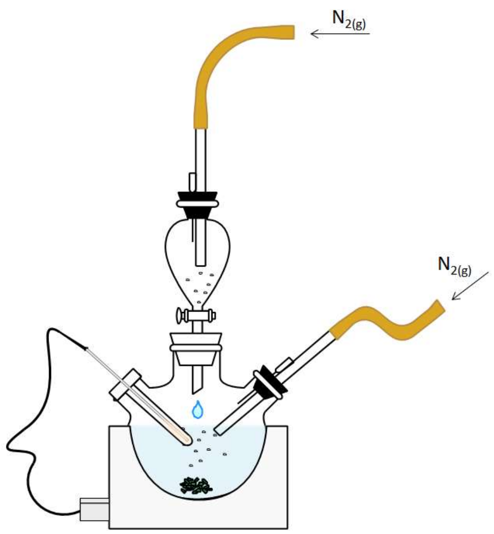

Figure S1. Schematic illustration of the experimental setup employed for the synthesis of Fe_3O_4 NPs and Au-Fe_3O_4 HNs.

Estimation of the concentration of Au NPs

The estimation of the Au NPs concentration was performed based on the Lambert and Beer law and on the assumption that the synthesized Au NPs consists of Au nanospheres of a single diameter. Considering that, the extinction cross section of a Au nanosphere, C_{ext}, can be expressed as:

$C_{ext} = \pi N_A D^2 Q_{ext}$,

where N_A is the Avogadro's number, and D and Q_{ext} stand by the diameter and extinction efficiency of the nanosphere, respectively, the measured absorbance A is given by:

$A = C_{ext}\, b\, C$,

where b is the optical path length of the cuvette (1 cm), and C is the concentration of Au NPs. The fitting of the measured extinction spectrum to spectra simulated with the Mie theory allows to determine D and Q_{ext} and therefore C_{ext}. The extinction spectrum of the synthesized Au NPs is shown in SI Figure 2, black curve. On the other hand, the red curve represents the simulated extinction efficiency spectrum of a D=57 nm Au nanosphere dispersed in water, which has a $Q_{ext} = 4.74$ at $\lambda = \lambda_{LSPR}$, that is $\lambda = 535$ nm, resulting in $C_{ext} = 2.9 \times 10^{14}$ cm^2/mol. Taking into account that A = 0.269

at λ = 535 nm, it turns that C = 9.2 x 10^{-16} mol/cm^3 or 9.2 x 10^{-13} mol/L, a value that can be expressed approximately as ~ 1 x 10^{-12} M.

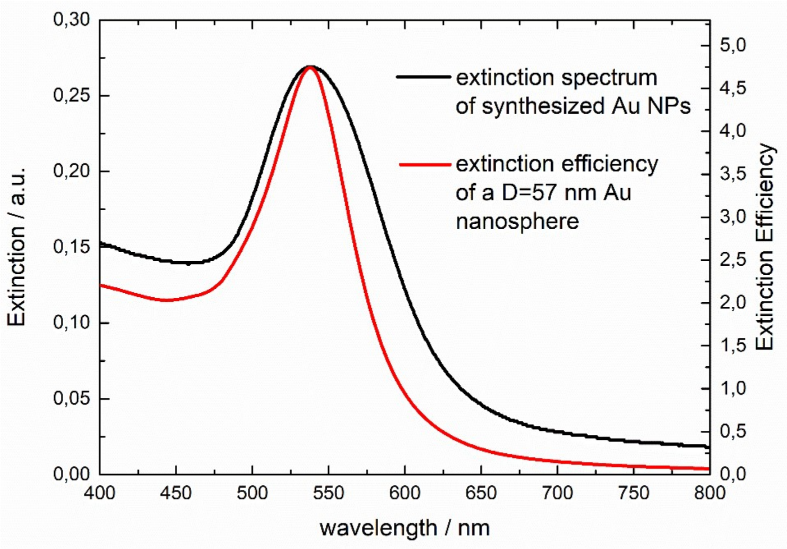

Figure S2. Extinction spectrum of the purified Au NPs (black curve) and simulated extinction efficiency spectrum of a D=57 nm Au nanosphere dispersed in water.

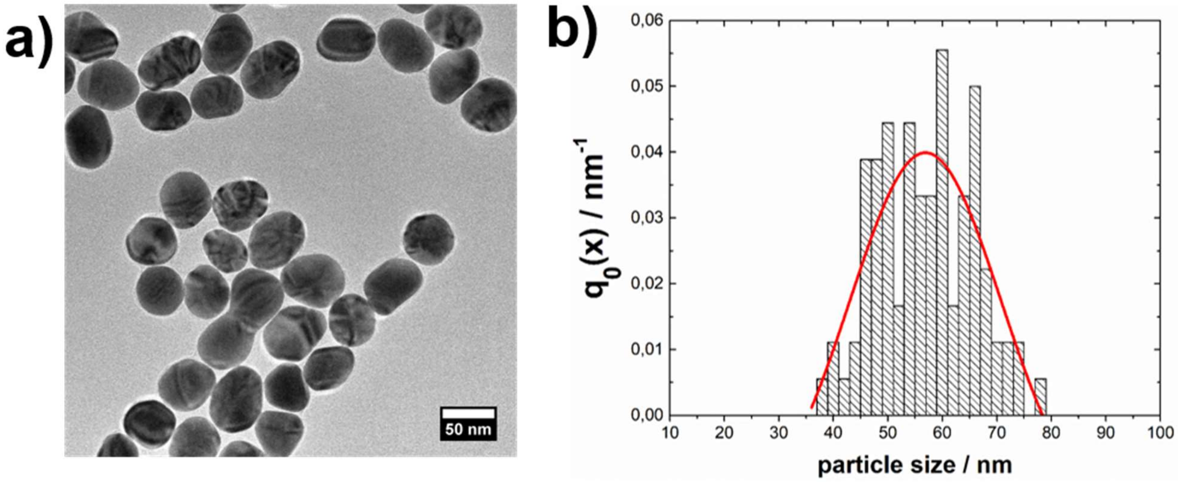

Figure S3. (a) Representative TEM image and (b) number density distribution of the size, q_0, of the Au NPs employed for the synthesis of the Au-Fe_3O_4 HNs. The red curve in panel (b) corresponds to a fit to a normal distribution centered at 57 nm.

Visual change in the color of the colloidal solution after the formation of Au-Fe_3O_4 HNs

The pink color of the Au NPs suspensions changes to a brown color after formation of the Au-Fe_3O_4 HNs.

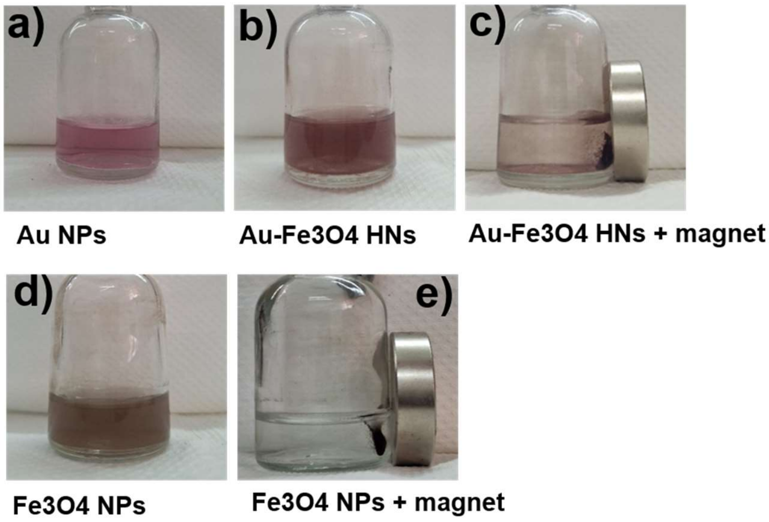

Figure S4. Photos of colloidal dispersions of (a) Au NPs, (b) Au-Fe_3O_4 HNs and (d) Fe_3O_4 NPs. Photos shown in panels (c) and (e) illustrates the magnetic response of the synthesized materials.

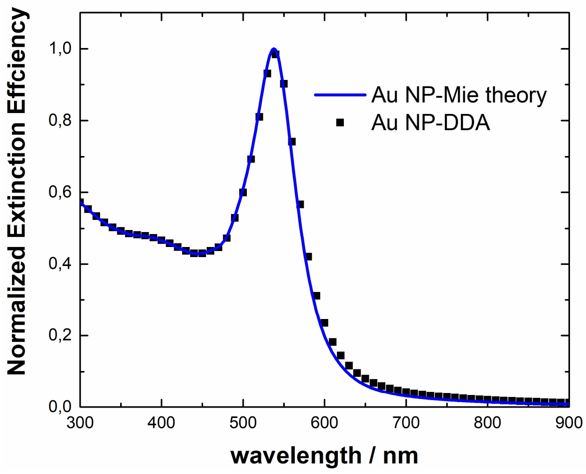

Figure S5. Comparison between the normalized extinction efficiency spectra of a 57 nm diameter Au nanosphere immersed in water calculated through the Mie theory (blue curve) and the

approximated DDA method (black dots). The very good agreement between both spectra indicates that a tolerable error is reached when using an inter-dipole distance of 1 nm to model the Au nanosphere.

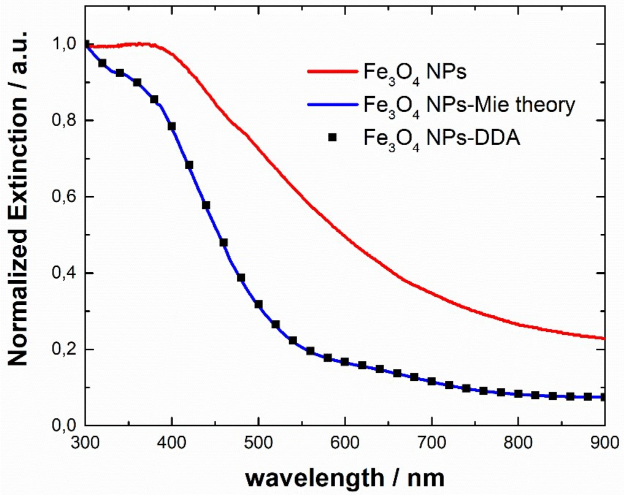

Figure S6. Comparison between the normalized extinction efficiency spectra of a 30 nm diameter Fe_3O_4 nanosphere immersed in water calculated through the Mie theory (blue curve) and the approximated DDA method (black dots), where it can be appreciated the high accuracy of the later.

The very good agreement between both spectra indicates that a tolerable error is reached when using an inter-dipole distance of 1 nm to model the Fe_3O_4 nanosphere. In addition, the experimental extinction spectrum of the synthesized Fe_3O_4 NPs (see Figures 2a and 2b) is shown in red curve. The profiles of the simulated spectra and of the experimental spectrum measured for the Fe_3O_4 NPs aqueous dispersion are quite similar. The larger experimental extinction values with respect to the simulated ones are attributed to larger scattering contributions from the bigger Fe_3O_4 NPs of the distribution (see Figure 2b) as well as to aggregates of Fe_3O_4 NPs that might be dispersed in solution.

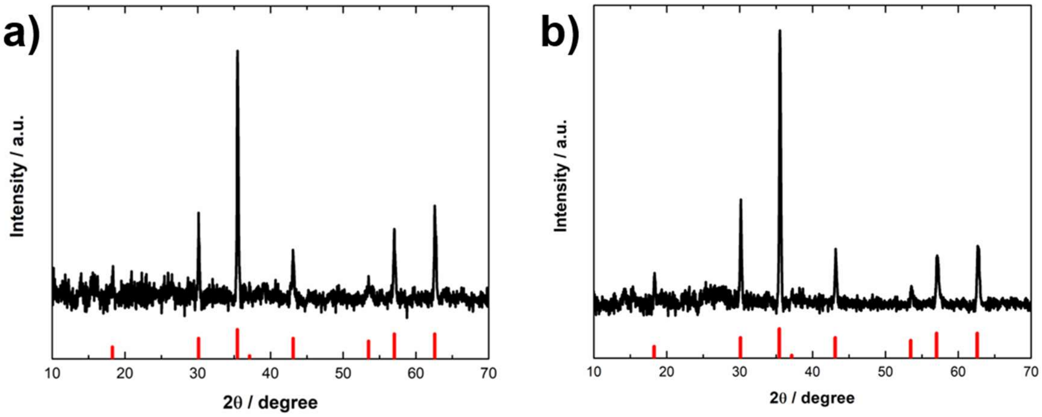

Figure S7. XRD pattern of Fe_3O_4 NPs synthesized with R=2, Fe(II) concentration 5x10^{-5} M, and addition rate of H_2O_2 solution of (a) 100 mL/s and (b) 10 mL/s. The reference XRD pattern of Fe_3O_4 (JCPDS 01-089-0691) is shown in the lower part in red bars.
